# Mesoporous CLEAs-silica composite microparticles with high activity and enhanced stability

**DOI:** 10.1038/srep14203

**Published:** 2015-09-16

**Authors:** Jiandong Cui, Shiru Jia, Longhao Liang, Yamin Zhao, Yuxiao Feng

**Affiliations:** 1 Research Center for Fermentation Engineering of Hebei, College of Bioscience and Bioengineering, Hebei University of Science and Technology, 26 Yuxiang Street, Shijiazhang 050018, P R China; 2 Tianjin Key Laboratory of Food-Biotechnology, Tianjin University of Commerce, Beichen district, Tianjin 300134, P R China; 3 Key Laboratory of Industry Microbiology, Ministry of Education, Tianjin University of Science and Technology, 29 Thirteenth Street, Tai Da Development Area, Tianjin 300457, P R China

## Abstract

A novel enzyme immobilization approach was used to generate mesoporous enzymes-silica composite microparticles by co-entrapping gelatinized starch and cross-linked phenylalanine ammonia lyase (PAL) aggregates (CLEAs) containing gelatinized starch into biomemitic silica and subsequently removing the starch by α-amylase treatment. During the preparation process, the gelatinzed starch served as a pore-forming agent to create pores in CLEAs and biomimetic silica. The resulting mesoporous CLEAs-silica composite microparticles exhibited higher activity and stability than native PAL, conventional CLEAs, and PAL encapsulated in biomimetic silica. Furthermore, the mesoporous CLEAs-silica composite microparticles displayed good reusability due to its suitable size and mechanical properties, and had excellent stability for storage. The superior catalytic performances were attributed to the combinational unique structure from the intra-cross-linking among enzyme aggregates and hard mesoporous silica shell, which not only decreased the enzyme-support negative interaction and mass-transfer limitations, but also improved the mechanical properties and monodispersity. This approach will be highly beneficial for preparing various bioactive mesoporous composites with excellent catalytic performance.

Enzymes are highly effective and versatile biological catalysts that exhibit high chemo-, regio- and enantio-selectivity at ambient temperatures. As a result, there is significant interest in using enzymes as an alternative to conventional chemical catalysts^1^,^2^,^3^. However, most biocatalysts (free enzymes) display low operational stability and difficulties in recovery and reuse, leading to high cost and the contamination of the products^4^,^5^. Immobilization of enzymes is a promising technology that can overcome these limitations. The stability of enzymes can be improved by immobilization technology; furthermore, immobilized enzymes can easily be recovered. The main causes for stabilization of immobilized enzymes are prevention of subunit dissociation via multisubunit immobilization^6^, prevention of aggregation^7^, autolysis or proteolysis by proteases^8^, rigidification of the enzyme structure via multipoint covalent attachment^9^, and generation of favorable microenvironments^10^,^12^. However, immobilized enzymes are still considerably less effective than free enzymes due to several drawbacks, such as the strong enzyme-support interaction, the enhanced diffusion limit, which are harmful to their catalytic performances. Therefore, the development of novel immobilized enzymes is still a major research interest. To date, numerous efforts have been devoted to the development of the immobilization of enzymes. Among various immobilization methods, cross-linked enzyme aggregates (CLEAs) seem to be a promising method for enzyme immobilization^11^,^13^. The enzyme is immobilized with high stability and high volume activity by cross-linking the physical enzyme aggregates^13^,^14^,^15^. Unfortunately, CLEAs may still be considered too soft for many industrial applications. Small CLEAs can form clumps during centrifugation and filtration treatments, which would hamper CLEAs to disperse again in solution, leading to low catalytic efficiency^16^,^17^,^18^,^19^. Considering these drawbacks to the industrial application of CLEAs, an innovative approach is required. Biomimetic silicification technology has recently been demonstrated to be a simple and effective method for enzyme immobilization via the *in situ* entrapment of enzymes^20^,^21^. Compared to other immobilization methods, the advantages of these biomimetic silicas include their mild processing conditions, low cost, and short preparation time, as well as excellent immobilization efficiency, high activity, and improved stability^22^,^23^. Successful biomimetic silicification of a broad range of enzymes, including D-amino acid oxidase^24^, dehydrogenases^25^, and carbonic anhydrase^26^, has been reported. However, the encapsulated enzymes in biomimetic silica also exhibited diffusion problems and limited recovery^21^,^25^.

To address the limitations associated with current immobilization technology, we utilized phenylalanine ammonia lyase (PAL) from *Rhodotorula glutinis* (*R.* g*lutinis*) as a model system, and reported a simple approach to preparing novel mesoporous CLEAs-silica composite microparticles (P-CLEAs-Si). As illustrated in Fig. 1, gelatinized starch and CLEAs containing the gelatinized starch were simultaneously encapsulated in biomimetic silica. The gelatinized starch was then removed by α-amylase treatment to form mesoporous CLEAs-silica composite microparticles. For comparison, conventional CLEAs, encapsulated PAL in biomimetic silica, and encapsulated CLEAs in biomimetic silica were also prepared, and named as CLEAs, PAL-Si, and CLEAs-Si, respectively. Compared to CLEAs and PAL-Si, P-CLEAs-Si exhibited excellent catalytic activity and stability. The superiority of P-CLEAs-Si could be due to the following: (1) the biomimetic silica coating increases the mechanical strength of CLEAs particles, which can protect CLEAs from the damage of mechanical stirring, and overcome the drawback of clumping of CLEAs due to separation during centrifugation or filtration. (2) The mesoporous structure of P-CLEAs-Si allows easier access of the substrate to the enzyme and excretion of the product, and decreases internal mass-transfer limitations. (3) CLEAs entrapped in biomimetic silica obstruct enzyme leaching, prevent structural deformation caused by heating and solvent corrosion, and enhance monodispersity of CLEAs to a certain extent. (4) P-CLEAs-Si could be easily recycled compared to CLEAs and PAL-Si due to increased particle size and mechanical strength.

## Results

### Synthesis and characterization of mesoporous CLEAs-silica composite microparticles

**Figure 1:**
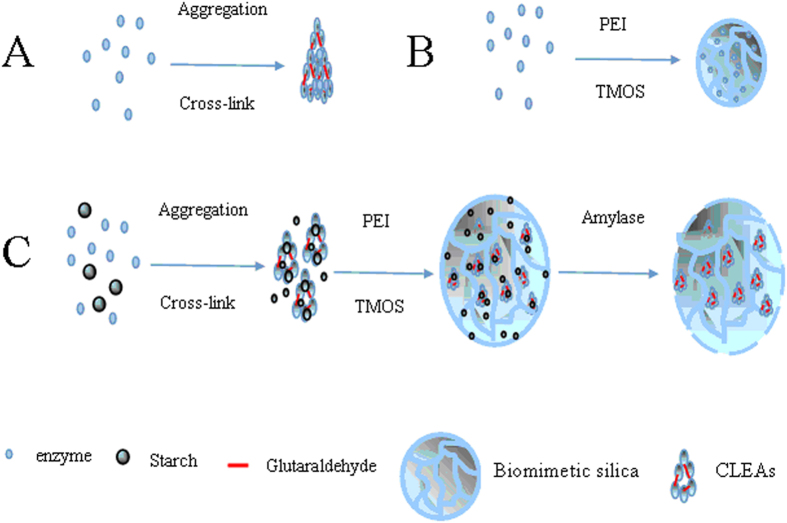
Schematic illustration of the synthesis strategy. (**A**) CLEAs, (**B**) PAL-Si, and (**C**) P-CLEAs-Si.

The synthesis of P-CLEAs-Si involved three main steps (Fig. 1C). First, PAL was co-aggregated with gelatinized starch and subsequently cross-linked with glutaraldehyde to obtain CLEAs containing gelatinized starch. Second, the resulting CLEAs and gelatinized starch were encapsulated simultaneously in biomimetic silica. Third, the starch in CLEAs and biomimetic silica was removed by adding α-amylase to form mesoporous CLEAs-silica composite microparticles. The advantages of using starch as a pore-forming agent are that it can be dissolved into the enzyme solution system with favorable homogeneity, and co-precipitated with enzymes by adding precipitant. In addition, the gelatinized starch cannot be cross-linked with glutaraldehyde, and can therefore be hydrolyzed into maltose and glucose, which can be washed away by water without any residues. Furthermore, residual starch can easily be examined by the I_2_–KI indicator.

**Figure 2:**
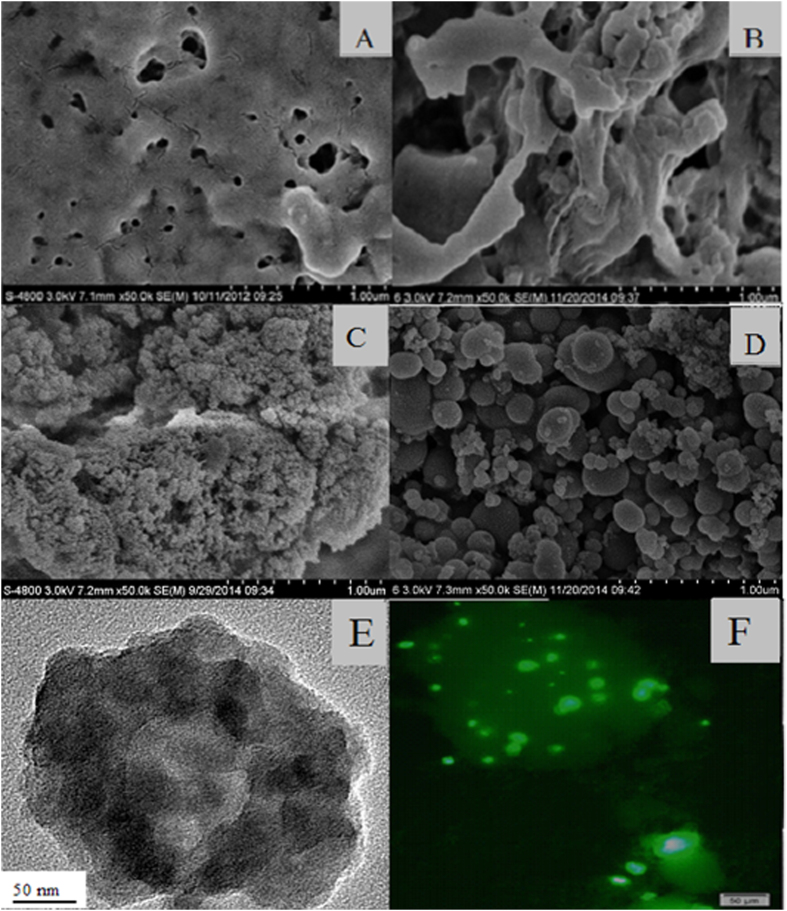
SEM images of (A) CLEAs, (B) CLEAs containing starch (after α-amylase treatment), (C) PAL-Si, (D) CLEAs-Si; TEM images of (E) P-CLEAs-Si; LCSM images of (E) P-CLEAs-Si with fluorescamine treatment.

Figure 2A illustrated the structure of CLEAs which was prepared by the conventional method. Apparently, the surface of CLEAs was relatively smooth, except for a few tiny pores. However, after removal of the gelatinized starch by α-amylase, several large porous structures of CLEAs were observed, due to the pore-forming function of starch (Fig. 2B). In addition, small aggregates of fused silica particles were observed in the structure of PAL-Si, which was prepared according to the conventional method (Fig. 2C). In contrast, the CLEAs-Si particles had a regular spherical shape of 100–500 nm and a relatively smooth surface (Fig. 2D). Furthermore, after encapsulation of CLEAs in biomimetic silica, CLEAs-Si particles were relatively dispersed, larger in diameter, and had different surfaces compared to PAL-Si particles. The above results showed that the structure of the conventional CLEAs is different from that of PAL-Si and CLEAs-Si, which indicating different catalytic properties. We estimated that the conventional CLEAs with tiny pores had major limitations in substrate diffusion, which restricted their entry into the inner core of CLEAs. However, CLEAs with large pores (treated by α-amylase) not only decreased the steric hindrance, but also increased the catalytic specific surface of CLEAs particles. In addition, the small particle size of PAL-Si resulted in difficulty to recover them from the reaction medium. Furthermore, the aggregation of fused silica particles may lower total surface area and increase mass transfer resistance, leading to inferior catalytic efficiency.

**Figure 3:**
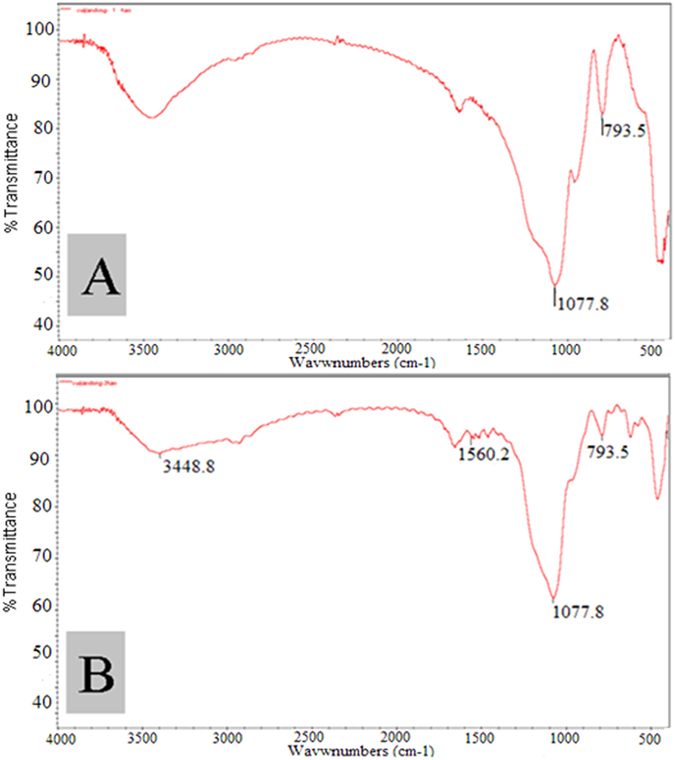
FT-IR spectra analysis. (**A**) Biomimetic silica and (**B**) P-CLEAs-Si.

Figure. 2E showed TEM images of P-CLEAs-Si, indicating that the CLEAs particles are encapsulated in biomemitic silica. Encapsulation of CLEAs in biomemitic silica could be further confirmed by fluorescence micrograph using fluorescein isothiocyanate (FITC) labeled CLEAs since the labeled CLEAs showed a typical green fluorescence image (Fig. 2F). FTIR spectrum of lyophilized P-CLEAs-Si revealed the characteristic absorptions of SiO_2_ at 1107.8 cm^−1^ and 793.5 cm^−1^, demonstrating successful preparation of the biomimetic silica (Fig. 3A,B). The bands at 1560.2 cm^−1^ in the FT-IR spectrum of CLEAs-Si could be attributed to -N-H stretching vibrations^27^, indicating encapsulation of CLEAs in the biomimetic silica (Fig. 3B). Moreover, the FTIR spectrum displayed a stronger absorbance at 3450 cm^−1^ corresponding to the absorbed water. CLEAs-Si entrapped significant amount of water, which helped to retain the three dimensional conformation of the PAL molecule. Taken together, we show that CLEAs were successfully encapsulated in biomimetic silica.

**Figure 4:**
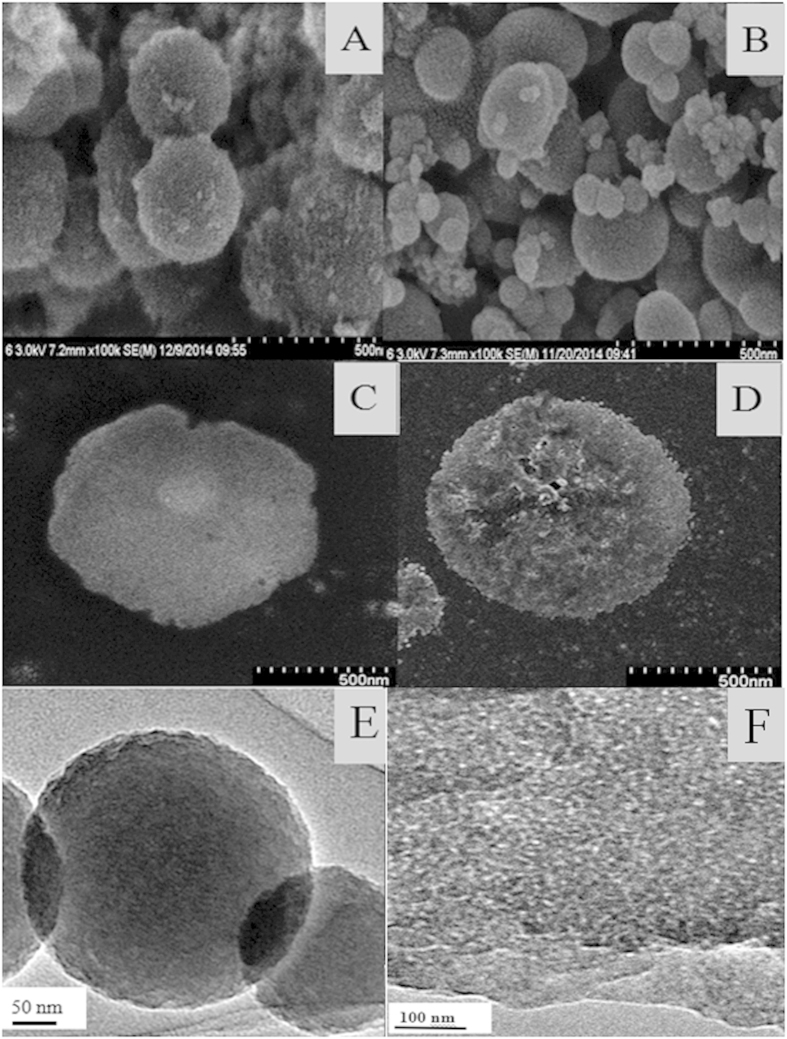
Particle analysis of immobilized PALs by SEM analysis. SEM images of (**B**,**C**) CLEAs-Si, (**A,D**) P-CLEAs-Si; TEM images of (**E**) P-CLEAs-Si, TEM images of (**F**) ultramicrotomed P-CLEAs-Si.

**Figure 5:**
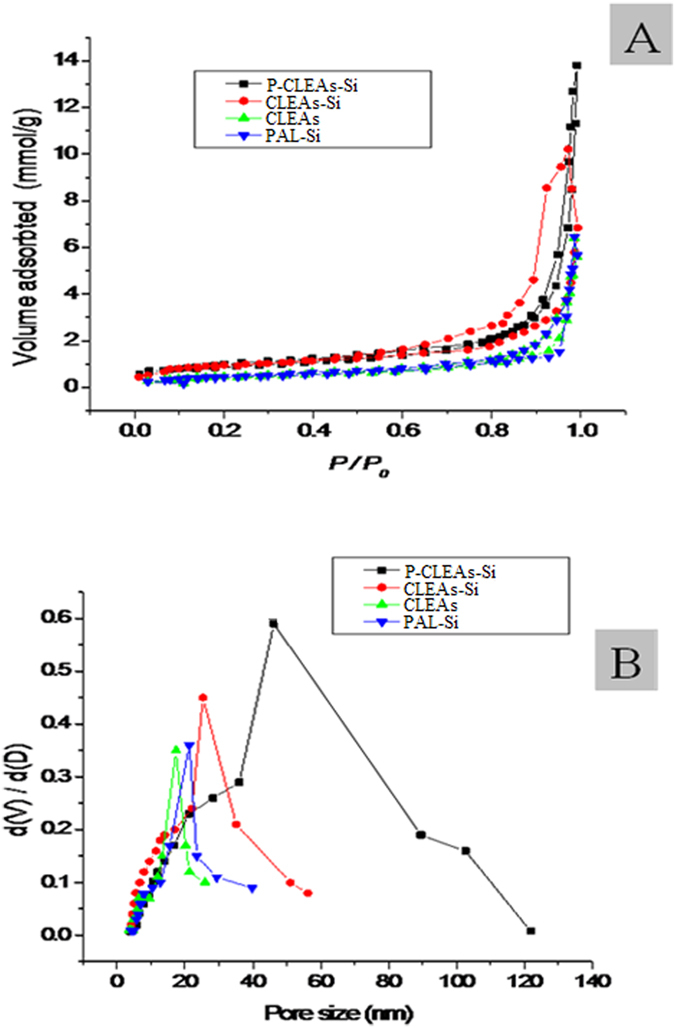
(**A**) N_2_ adsorption-desorption isotherms and (**B**) pore size distribution curves of CLEAs, PAL-Si, CLEAs-Si, and P-CLEAs-Si samples.

SEM images showed that P-CLEAs-Si were compact spherical particles (Fig. 4A). A dynamic light scattering (DLS) experiment revealed the average diameter of P-CLEAs-Si was about 500 nm with a lower polydispersity than that of standard CLEAs (Figure S1), which translates to higher catalytic efficiency. Generally, diffusion limitations cause low substrate accessibility, which lead to low catalytic efficiency of CLEAs and biomimetic silica^24^,^28^. We therefore utilized gelatinized starch as a pore-forming agent to decrease diffusion limitations. The porosity of P-CLEAs-Si was examined by SEM, TEM and a surface area and pore size analyzer. In contrast to P-CLEAs-Si, which possessed a blurred outlines and rough surfaces (Fig. 4A,D), CLEAs-Si had distinct outlines and smooth surfaces (Fig. 4B,C). This suggests an improvement in P-CLEAs-Si permeability. A TEM image showed that individual P-CLEAs-Si particles were produced, with no aggregation observed. These particles retained the spherical shape (Fig. 4E). Furthermore, the porosity of P-CLEAs-Si was examined by TEM analysis of ultramicrotomed (~90 nm thin slices) samples. TEM images indicated that P-CLEAs-Si contains well-defined mesoscale pores (Fig. 4F). The coarse surface and porous structures of P-CLEAs-Si can be attributed to the pore-forming function of starch. To further test the porous structures of P-CLEAs-Si, we investigated the pore size distribution in all the immobilized PAL particles by nitrogen gas adsorption/desorption analysis. All samples displayed a type IV isotherm with H_1_ hysteresis, which is typical of mesoporous structures^29^,^30^ (Fig. 5A). Pore size distribution analysis (Fig. 5B) revealed that the pore sizes of CLEAs (17 nm) and PAL-Si sample (20 nm) were smaller than those of CLEAs-Si (26 nm). However, the pore size of P-CLEAs-Si (46 nm) was considerably larger than that of all other samples (Table 1). Starch created larger pores in biomimetic silica and CLEAs following α-amylase digestion. BET surface area of CLEAs-Si increased from 268 cm^2^/g to 313 cm^2^/g after starch digestion (Table 1). This further confirms that the porosity of P-CLEAs-Si is due to the pore-forming function of gelatinized starch. The porous structure and the increased specific surface of P-CLEAs-Si particles are critical for ensuring high catalytic efficiency, due to an increase in mass substrate transfer for interaction with the interior enzymes of CLEAs.

**Figure 6:**
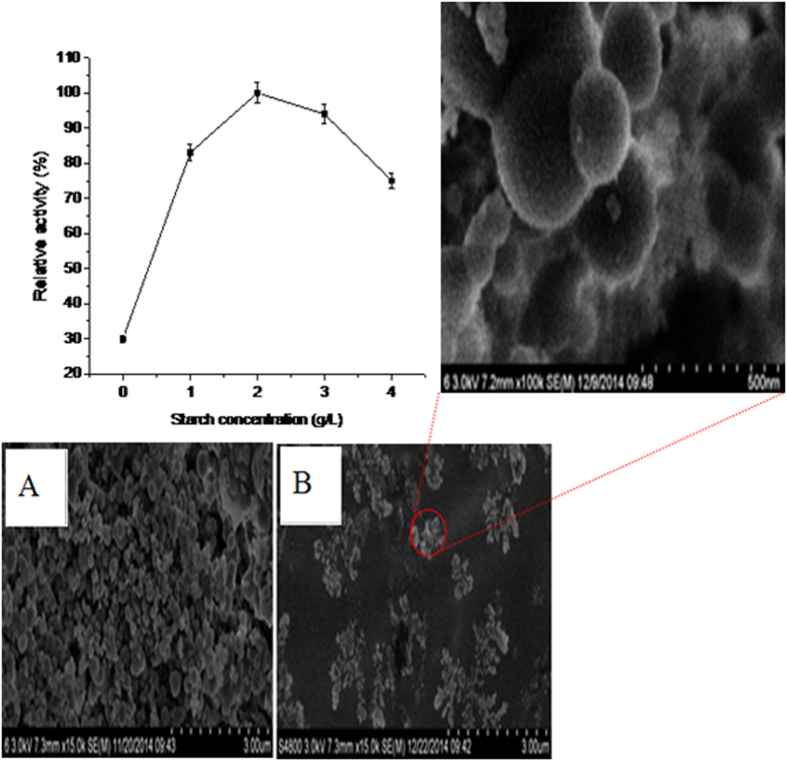
Effect of starch concentration on the PAL activity. SEM images of (**A**) CLEAs-Si with 2 g/L gelatinized starch treatment; (**B**) CLEAs-Si with 4 g/L gelatinized starch treatment; error bars show standard deviations for triplicate measurements.

Generally, porous structures can be controlled during preparation. In order to validate the effect of gelatinized starch on pore formation in P-CLEAs-Si, their activity was measured after the addition of different starch concentrations (Fig. 6). Starch generally occupies some of the inner space of CLEAs and biomimetic silica during P-CLEAs-Si preparation; however, the number of these spaces, which become pores after starch removal, was proportional to starch concentration. Consequently, the amount of pores increased with increasing starch concentrations, which resulted in better mass-transfer conditions and allowed easier access of substances to the active sites of enzymes. The highest PAL activity in P-CLEAs-Si was observed with a starch concentration of 2 g/L, and the resulting P-CLEAs-Si had regular and intact porous spherical particles at this concentration (Fig. 6A). A higher starch concentration of 4 g/L was not ideal for the formation of homogeneous starch and free enzyme mixtures, due to over-viscous of the gelatinized starch during preparation. This led to irregular P-CLEAs-Si particle and pore sizes, CLEA leaching due to particle rupture, and reduced PAL activity (Fig. 6B).

### Activity of P-CLEAs-Si

Based on the enzyme amounts in solution before and after the immobilization measured with the Bradford method, similar immobilization efficiency was obtained for all immobilization enzyme particles, and the PAL loadings in conventional CLEAs, PAL-Si, and P-CLEAs-Si were 79%, 85% and 83%, respectively. Besides, the kinetic parameters of free PAL, conventional CLEAs, PAL-Si, CLEAs-Si, and P-CLEAs-Si were determined by calculating the initial rates at various substrate concentrations. Table 2 shows the *V*_*max*_, *K*_*m*_, and *V*_*max*_/*K*_*m*_ values for the five forms of PAL. P-CLEAs-Si displayed similar *K*_*m*_ and *V*_*max*_ values as native PAL, indicating that it retained most of its original activity. Moreover, *K*_*m*_ value of P-CLEAs-Si was lower than that of CLEAs-Si. This could mainly be attributed to the porosity of P-CLEAs-Si, which possesses better mass-transfer conditions, thus allowing convenient transport of substrates into PAL active sites. However, compared to P-CLEAs-Si, the *V*_max_ and *V*_max_/*K*_*m*_ of PAL-Si, conventional CLEAs, and CLEAs-Si were significantly reduced, and this reduced catalytic efficiency (*V*_max_/*K*_*m*_) could be ascribed to the presence of irregular particles, fewer porous structures, and smaller surface areas.

The effect of reaction temperature and pH on the activity of all immobilized PAL samples was compared. The effect of reaction temperature on the activity of CLEAs, PAL-Si, CLEAs-Si, and P-CLEAs-Si was similar, and all demonstrated their highest activity at 50 °C (Figure S2A). Similar results were observed for the optimal pH, and all immobilized PAL exhibited the highest activity at pH 9 (Figure S2B). These findings might be due to the fact that a larger percentage of PAL aggregates is directly cross-linked and encapsulated in the biomimetic silica; therefore, the enzyme structure and microenvironment around the active site of PAL in the biomimetic silica was not dramatically changed and remained similar to that of the standard CLEAs.

### Stability of P-CLEAs-Si

**Figure 7:**
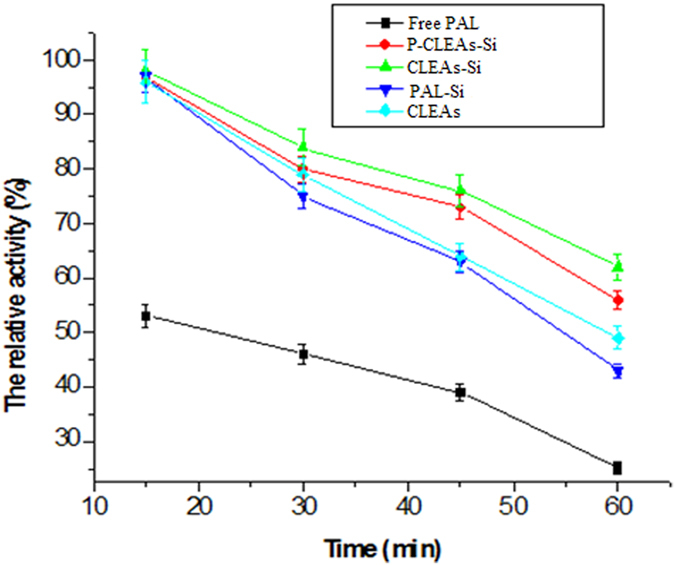
Thermal stability of free PAL, CLEAs, PAL-Si, CLEAs-Si, and P-CLEAs-Si at 60 °C; error bars show standard deviations for triplicate measurements.

**Figure 8:**
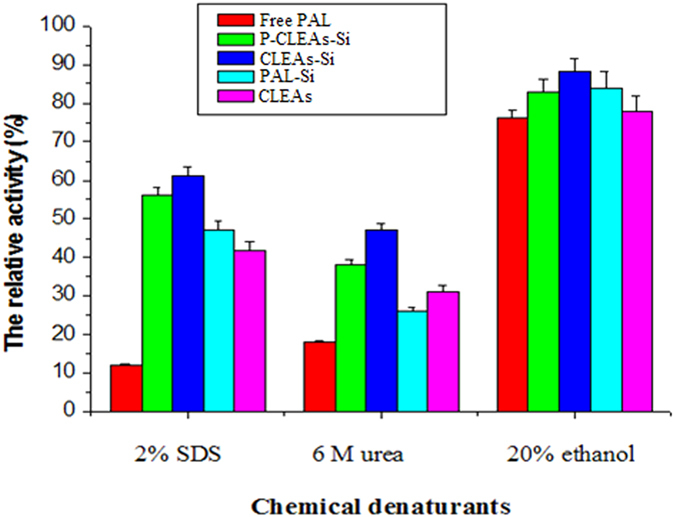
Stability of free PAL, CLEAs, PAL-Si, CLEAs-Si, and P-CLEAs-Si against different denaturants; error bars show standard deviations for triplicate measurements.

The stability of enzymes against high temperatures and chemical denaturants is critical for their industrial applications^31^. Therefore, the stability of native PAL, CLEAs, PAL-Si, CLEAs-Si, and P-CLEAs-Si against heating and chemical denaturants was compared. All immobilized PAL samples exhibited better thermal stability than free PAL, and the thermal stability of P-CLEAs-Si was better than that of both CLEAs and PAL-Si (Fig. 7). A similar phenomenon was also observed while assessing the stability of PAL against chemical denaturants (Fig. 8). P-CLEAs-Si retained 60% and 56% of its initial activity after 1 h incubation at 60 °C and in 2% SDS for 30 min, respectively; however, under the same conditions, conventional CLEAs only retained 51% and 42%, respectively. In addition, we examined the performance of free PAL and all immobilized PAL under variations in pH. As shown in Figure 3S, PAL-Si, CLEAs-Si, and P-CLEAs-Si were much more stable than the free PAL and CLEAs after incubating the system over a pH range between 3 and 11 for 1 h at 25 °C. At highly acidic condition (pH 3.0), free PAL and conventional CLEAs lost most of their activity, whereas P-CLEAs-Si still maintained 60% of its initial activity. The increased tolerance towards high temperature, extreme pH and chemical denaturants for P-CLEAs-Si may be due to the following reasons: (1) entrapment of water molecules in the biomimetic silica could help enzyme retain its structure and function; (2) the silica shell may provide a suitable microenvironment to reduce the deformation of PAL structures at high temperatures, extreme pH, and under harsh denaturants due to the retard of heat transfer and the denaturant corrosion^32^; (3) intra-cross-linking among enzyme aggregates improved the rigidity of the active conformation. In short, P-CLEAs-Si constructed unique dual protections derived from the intra-cross-linking among enzyme aggregates and hard porous silica shell.

**Figure 9:**
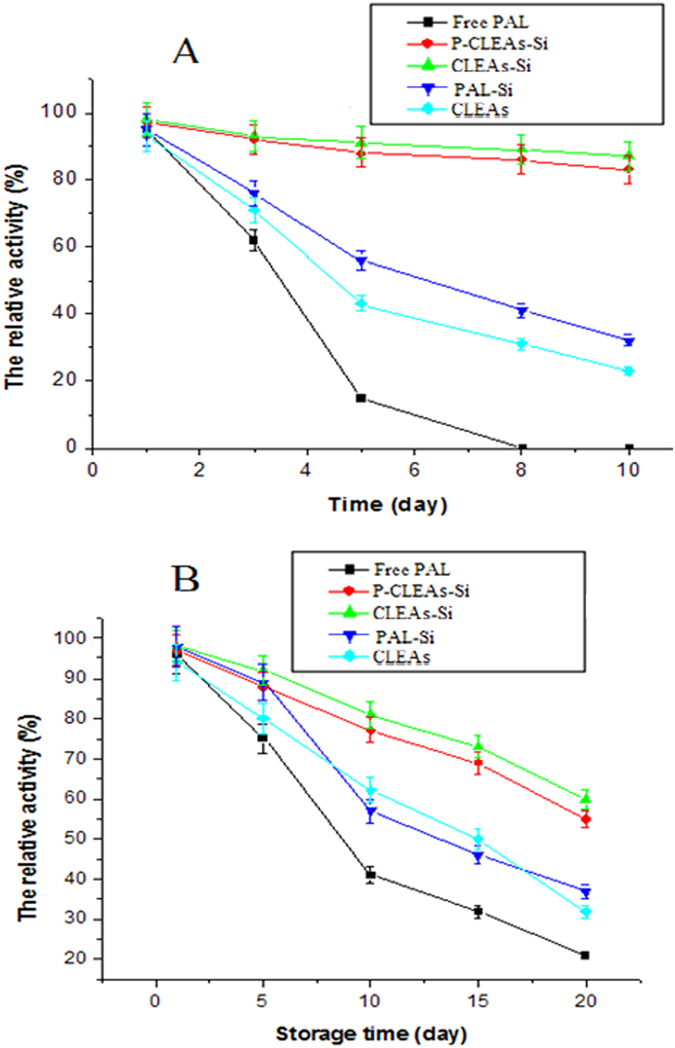
Mechanical stability (A) and storage stability (B) of free PAL, CLEAs, PAL-Si, CLEAs-Si, and P-CLEAs-Si; error bars show standard deviations for triplicate measurements.

In addition, we evaluated the stability of native PAL, CLEAs, PAL-Si, CLEAs-Si, and P-CLEAs-Si against mechanical damage and leaching by incubating them in aqueous solution under shaking conditions (200 rpm). Native PAL activity decreased considerably and total activity was lost by 8 days (Fig. 9A). Conventional CLEAs and PAL-Si lost most of their activity by 8 days, and retained only 40% and 30% of their initial activity, respectively, after 10 days of shaking. In contrast, P-CLEAs-Si retained 90% of its initial activity after 10 days of shaking. The fast decay of CLEAs and PAL-Si sample originated from the leaching of PAL molecule, which was detected in the supernatant. No significant P-CLEAs-Si leaching was observed after 10 days of shaking, and it still maintained intact spherical particles after 10 days of shaking as evidenced by TEM image (Figure S4). Furthermore, CLEAs were still encapsulated in the biomimetic silica after 10 days of shaking as evidenced by LCSM image (Figure S5). All the results demonstrated that P-CLEAs-Si was more stable against mechanical damage and leaching than conventional PAL-Si and CLEAs. The stabilization of PAL activity in P-CLEAs-Si might be due to intra-cross-linking of PAL molecules in the form of cross-linked clusters, which effectively prevents the denaturation of the enzyme molecules. Moreover, the protective role of the hard silica shell may also contribute to the stability of P-CLEAs-Si. Therefore, this approach of CLEAs entrapped in biomimetic silica materials may be potentially useful for industrial biocatalysis applications, since the enzyme is protected from mechanical damage caused by shaking or stirring during industrial production. In addition, the activity of P-CLEAs-Si decreased at a much slower rate compared to that of the free PAL, PAL-Si, and CLEAs (Fig. 9B). P-CLEAs-Si retained about 60% of its initial activity at 25 °C for 20 days storage; however, conventional CLEAs and PAL-Si only retained 40% and 35% of their initial activity, respectively, under the same conditions. Free PAL lost most of its activity during the same storage period. We reasoned that the excellent storage stability of P-CLEAs-Si was due to the entrapment of the cross-linked PAL molecules in the biomimetic silica, which prevented their escape.

**Figure 10:**
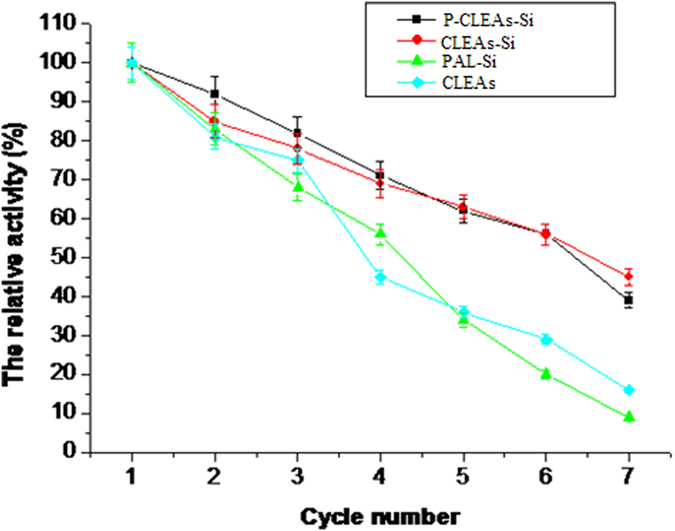
Stability of P-CLEAs-Si during repeat cycles; error bars show standard deviations for triplicate measurements.

For any industrial application, the reusability of immobilized enzymes is a key factor for its cost-effective use^33^. Therefore, the reusability of all immobilized PAL for repeated batch biotransformation of trans-cinnamic acids to L-phenylalanine was evaluated. As shown in Fig. 10, P-CLEAs-Si could be reused for 3 cycles without dramatic activity loss and still retained 45% of its initial activity after the 7 cycles. However, after the 7^th^ cycle, conventional CLEAs and PAL-Si only retained approximately 20% and 10% activity, respectively. Taken together, all of these results indicate that PAL immobilized using the P-CLEAs-Si method was effective in improving the enzyme’s catalytic performance and achieving long-term stabilization of enzyme activity under continuous recycling.

## Discussion

In this study, an immobilized enzyme was generated using a novel approach involving co-entrapment of gelatinized starch and CLEAs co-aggregated with starch into biomimetic silica and subsequent removal of the starch in CLEAs and biomimetic silica by α-amylase. The resulting mesoporous CLEAs-silica composite microparticles displayed high activity and excellent stability, which could be ascribed to the synergic effect generated from intra-cross-linking between enzyme aggregates and the hard porous silica shell. This unique structure of mesoporous CLEAs-silica composite microparticles not only decreased the undesired enzyme-support interactions and the mass transfer limitation, but also improved its mechanical properties and monodispersity. Therefore, this approach has high potential for preparing various bioactive porous composites with improved catalytic performance for use in biocatalysis and biomedicine.

## Methods

### Chemicals

Glutaraldehyde was obtained from Sigma-Aldrich Inc. (St. Louis, MO, U.S.A.). Tetramethoxysilane (TMOS) and polyethyleneimine (PEI, 1.8 kDa) were purchased from International Aladdin Reagent Inc. (Shanghai, China). L-Phenylalanine was obtained from Beijing Chemical Reagent Company (Beijing, China). Starch (from corn, 350–750 kDa) and all other reagents used were of analytical grade.

### Production and purification of PAL from *R. glutinis*

The *R. glutinis* strain (CICC 20030) used throughout this study was obtained from the China Center of Industrial Culture Collection (CICC, Beijing, China). The strain was cultivated according to a previously described method^34^. The cells were harvested by centrifugation of the cell-suspension at 3000 × g. All subsequent procedures were carried out in an ice-bath. The pellets were resuspended in 25 mM Tris-HCI buffer (pH 8.8) and glass beads were used to disrupt the cell suspension. The extract was centrifuged at 10,000 × g for 30 min and the supernatant was brought to 55% ammonium sulfate saturation by slowly adding solid (NH_4_)_2_SO_4_ while stirring for 30 min, and centrifuged (10,000 × g). The precipitate was redissolved in 25 mM Tris-HCI buffer (pH 8.8), and centrifuged (10,000 × g). The supernatant (10 ml) was then loaded onto a Sepharose 4B gel column (Beijing DingGuo ChangSheng Biotechnology Co., Ltd, China). The active fractions were pooled and applied onto a DEAE-Sephacel column pre-equilibrated with 25 mM Tris-HCI buffer (pH 8.8). The adsorbed enzyme was eluted with a linear gradient of 75 ml of the same buffer containing 300 mM NaCl. The active fractions with PAL activity were pooled, and the resulting enzyme solutions were desalted by dialysis. The purity of the enzyme was confirmed by sodium dodecyl sulfate-polyacrylamide gel electrophoresis (SDS-PAGE).

### Preparation of CLEAs, PAL-Si, CLEAs-Si, and P-CLEAs-Si

CLEAs were prepared as previously reported^35^. For PAL-Si, a freshly prepared solution of 1 M TMOS in 1 mM HCl at room temperature was used as the source of silicic acid. The purified PAL solution (final protein concentration of 3 mg/ml, 10 U enzyme activity) and 6 mg/ml PEI were mixed in 25 mM potassium phosphate buffer (pH 6.8) at room temperature. The encapsulation reaction was initiated by the addition of 1 M hydrolyzed TMOS. The solution turned turbid within 5 min due to the formation of silica and the precipitate was collected via centrifugation at 10000 × g for 5 min. The precipitated biomimetic silica was washed three times with deionized water, and re-suspended in 25 mM Tris-HCl buffer, pH 8.8, and stored at 4 °C.

For CLEAs-Si preparation, 20 mg CLEAs (10 U enzyme activity) and 6 mg/ml PEI were mixed in 25 mM potassium phosphate buffer (pH 6.8) at room temperature. Afterwards, 10 μl of 1 M hydrolyzed TMOS was added to the mixture and the resulting solution was given a gentle mix and left unstirred for 5 min. The precipitated biomimetic silica containing CLEAs was collected by centrifugation. After washing three times with deionized water, the biomimetic silica was re-suspended in 25 mM Tris-HCl buffer, pH 8.8, and stored at 4 °C.

For P-CLEAs-Si preparation, gelatinized starch (2 g/L) and purified PAL solution (final proteins concentration of 3 mg/ml, 10 U enzyme activity) were mixed, and ammonium sulfate was added to a final 40% ammonium sulfate saturation under gentle stirring at 4 °C for 1 h. Glutaraldehyde (25% v/v) was then added to yield a final concentration of 0.2% (v/v) and stirred for 2 h at 4 °C. The suspension was centrifuged at 10,000 × g for 10 min at 4 °C. The resultant CLEAs (CLEAs of co-precipitation with enzyme and starch) were washed three times with deionized water, and stored at 4 °C. Secondly, the resultant CLEAs (20 mg, 10 U enzyme activity), the gelatinized starch (2 g/L), and 6 mg/ml PEI were mixed in 25 mM potassium phosphate buffer (pH 6.8) at room temperature. The resultant mixtures were mixed with 10 μl of 1 M hydrolyzed TMOS, and after 5 min, the mixtures were centrifuged for 5 min at 10,000 × g at 4 °C. The precipitates were washed three times with deionized water and re-suspended in 25 mM Tris-HCl buffer (pH 7.0). α-amylase (1 ml) was added and the mixture was incubated at room temperature. I_2_-KI indicator (contained 1% I_2_ and 2% KI) was added to the resultant mixtures to detect starch hydrolyzation^28^. After centrifugation, the insoluble P-CLEAS-Si was washed with distilled water three times and finally resuspended in 25 mM Tris-HCl buffer, pH 8.8, and stored at 4 °C.

### Activity assay

The enzyme activity of free PAL and immobilized PAL was determined based on a modified procedure^36^. A number of enzyme samples were added to a reaction medium consisting of 25 mM Tris-HCl buffer solution (pH 8.8) supplemented with 50 mM L-Phenylalanine. The resultant reaction medium was incubated at 30 °C for 20 min. The reaction was terminated by addition of 6 M HCl. After centrifugation, the rate of formation of trans-cinnamic acids was determined by measuring the increase in A_278_ _nm_ with a 2800 H spectrophotometer (Unicoi Instrument Co., Ltd. Shanghai). One unit of PAL activity was defined as the amount of enzyme required to convert one μmol of L-Phenylalanine to trans-cinnamic acids per minute. The activity assay of immobilized enzymes was detected by the same procedure as described above. The PAL immobilization efficiency was calculated using the following equation:


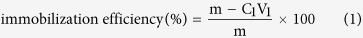


where m (mg) represents the mass of PAL initially added to the solution, C_1_ (mg/ml) represents the PAL concentration of the supernatant, and V_1_ (ml) represents the volume of the supernatant.

### Structural Characterization

SEM (JEOL JSM6700), TEM (JEOL JEM2100 and Philips CM120 BioTWIN), and laser particle size analyzer (Malven S-90) were used to examine the particle morphologies of CLEAs, CLEAs-Si, PAL-Si, and P-CLEAs-Si. The ultramicrotomed samples of P-CLEAs-Si were sliced to a thickness of 50~90 nm. Confocal laser scanning microscopy (CLSM) was used to investigate the distribution of CLEAs. Prior to observation, PALs were mixed with FITC solution (50 mg/ml, FITC in acetone) for 3 min to form a highly fluorescent product by the reaction between primary amines in proteins and fluorescamine^37^. Modified FITC labeled PALs were then immobilized. CLSM observation was performed with a Leica TCS SP5 microscope (Leica Camera AG, Germany). The samples were excited at 390 nm and FITC fluorescence was detected between 460 and 480 nm. N_2_ adsorption isotherms were obtained on a Beckman coulter SA3100 analyzer at 77 K. Specific surface areas and pore diameter distribution were calculated using Brunauer-Emmett-Teller (BET) and Barrett–Joyner–Halenda (BJH) models, respectively, based on the adsorption isotherm. The FTIR spectra of P-CLEAS-Si were obtained using a NEXUS870 infrared spectrometer (Thermo Nicolet Corporation, Madison, WI) using the standard KBr disk method.

### Measurement of kinetic parameters

To measure the kinetic parameters (*K*_*m*_ and *V*_*max*_) of free enzyme, CLEAs, PAL-Si, CLEAs-Si, and P-CLEAs-Si, initial velocities were measured with increasing L-Phenylalanine concentrations from 5.0 mM to 150 mM. *K*_*m*_ and *V*_*max*_ were calculated using the Lineweaver-Burk equation based on computed linear regression calculations.

### The stability of free PAL, CLEAs, PAL-Si, CLEAs-Si, and P-CLEAs-Si

The thermal stability of free PAL, CLEAs, PAL-Si, CLEAs-Si, and P-CLEAs-Si in aqueous solution was determined by incubating enzyme samples in 25 mM Tris-HCl buffer solution (pH 8.8) at 60 °C for 15–60 min, and the enzyme samples were taken out at the indicated time points. The residual activity of each enzyme sample was measured. The relative activity was calculated from the ratio of the residual activity to the initial activity of each sample. The stability of free PAL, CLEAs, CLEAs-Si, and P-CLEAs-Si against different denaturants was tested. The denaturing solutions consisted of urea (6 M), sodium dodecyl sulfate (SDS, 2%, w/v), or ethanol (20%, v/v) in 25 mM Tris-HCL buffer (pH 8.8). Each analysis lasted 30 min at 30 °C. Enzyme samples were then taken out and the residual activities were measured. The storage stability of free PAL, CLEAs, CLEAs-Si, and P-CLEAs-Si were determined by incubating enzyme samples in 25 mM Tris-HCl buffer solution (pH 8.8) at 25 °C. At different storage times, enzyme samples were separated and washed with distilled water. The residual PAL activity in these immobilized enzyme and free enzyme samples were determined. In addition, the free PAL, CLEAs, CLEAs-Si and P-CLEAs-Si were immersed in 25 mM Tris-HCl buffer solution (pH 8.8) at room temperature and shaken at 200 rpm for a certain time to detect mechanical stability and stability against leaching. Then the enzyme samples were taken out and centrifuged at each time point, the PAL activity in these immobilized enzyme and the supernatant liquid were measured, respectively.

### Recycling of CLEAs, PAL-Si, CLEAs-Si, and P-CLEAs-Si

The recycling ability of immobilized PAL for biotransformation of trans-cinnamic acids to L-Phenylalanine was evaluated. The reaction system (1.0 ml) consisted of 50 mg of immobilized PAL in 1 ml of 10 g/L trans-cinnamic acid, and 25% ammonium hydroxide (pH 10.5). Each cycle lasted for 2 h at 30 °C, followed by separation of the immobilized PAL from the reactants by centrifugation. The clear supernatant was then slowly decanted. After thoroughly washing with deionized water, the biocatalysts were re-used with freshly charged buffer and reactant for subsequent recycle runs under the same reaction conditions. The residual PAL activity of each cycle was calculated by taking the enzyme activity of the first cycle as 100%.

## Additional Information

**How to cite this article**: Cui, J. *et al.* Mesoporous CLEAs-silica composite microparticles with high activity and enhanced stability. *Sci. Rep.* **5**, 14203; doi: 10.1038/srep14203 (2015).

## Supplementary Material

Supplementary Information

## Figures and Tables

**Table 1:** The surface characteristics of CLEAs, PAL-Si, CLEAs-Si, and P-CLEAs-Si; error bars show standard deviations for triplicate measurements.

Sample	S_BET_ (cm^2^/g)	Pore volume (cm^3^/g)	Pore diameter (nm)
P-CLEAs-Si	313 ± 8.6	0.59 ± 0.02	46.12 ± 4.6
CLEAs-Si	268 ± 7.1	0.45 ± 0.02	25.34 ± 3.5
CLEAs	198 ± 6.2	0.35 ± 0.01	17.43 ± 2.6
PAL-Si	224 ± 6.8	0.39 ± 0.01	20.15 ± 3.1

**Table 2:** Comparison of kinetic parameters of free PAL, CLEAs, PAL-Si, CLEAs-Si, and P-CLEAs-si; error bars show standard deviations for triplicate measurements.

Enzyme	*K*_*m*_ (mM)	*V*_*max*_ (mM/mL·min)	*V*_*max*_*/K*_*m*_
Free PAL	0.56 ± 0.02	26.72 ± 3.6	47.71 ± 4.7
CLEAs	0.75 ± 0.03	16.54 ± 2.5	22.05 ± 3.3
P-CLEAs-Si	0.59 ± 0.02	25.68 ± 3.5	43.52 ± 4.2
PAL-Si	1.06 ± 0.05	12.85 ± 2.3	12.12 ± 2.3
CLEAs-Si	2.89 ± 0.5	4.82 ± 1.1	1.67 ± 0.07

## References

[b1] Panke S., Held M. & Wubbolts, M. Trends and innovations in industrial biocatalysis for the production of fine chemicals. Curr. Opin. Biotechnol. 15, 272–279 (2004). 15357999 10.1016/j.copbio.2004.06.011

[b2] Andexer J. N., Langermann J. V., Kragl U. & Pohl M. How to overcome limitations in biotechnological processes-examples from hydroxynitrile lyase applications. Trend Biotechnol. 27, 599–608 (2009). 10.1016/j.tibtech.2009.07.005 19716614

[b3] Dicosimo R., McAuliffe J., Poulose A. J. & Bohlmann G. Industrial use of immobilized enzymes. Chem. Soc. Rev. 42, 6347–6474 (2013). 10.1039/c3cs35506c 23436023

[b4] Cowan D. A. & Fernandez-Lafuente, R. Enhancing the functional properties of thermophilic enzymes by chemical modification and immobilization. Enzyme Microb. Tech. 49, 326–346 (2011). 10.1016/j.enzmictec.2011.06.023 22112558

[b5] Liese A. & Hilterhaus L. Evaluation of immobilized enzymes for industrial applications. Chem. Soc. Rev. 42, 6236–6249 (2013) 23446771 10.1039/c3cs35511j

[b6] Fernandez-Lafuente, R. Stabilization of multimeric enzymes: Strategies to prevent subunit dissociation. Enzyme Microb. Tech. 45, 405–418 (2009).

[b7] Mateo C., Palomo J. M., Fernandez-Lorente G., Guisan J. M. & Fernandez-Lafuente R. Improvement of enzyme activity, stability and selectivity via immobilization techniques. Enzyme Microb. Tech. 40, 1451–1463 (2007).

[b8] Rodrigues R. C., Ortiz C., Berenguer-Murcia Á., Torres R. & Fernández-Lafuente R. Modifying enzyme activity and selectivity by immobilization. Chem. Soc. Rev. 42, 6290–6307 (2013). 23059445 10.1039/c2cs35231a

[b9] Hernandez K. & Fernandez-Lafuente R. Control of protein immobilization: Coupling immobilization and site-directed mutagenesis to improve biocatalyst or biosensor performance. Enzyme Microb. Tech. 48, 107–122 (2011). 10.1016/j.enzmictec.2010.10.003 22112819

[b10] Iyer P. V. & Ananthanarayan L. Enzyme stability and stabilization-Aqueous and non-aqueous environment. Process Biochem. 43, 1019–1032 (2008).

[b11] Cao L., van Rantwijk F. & Sheldon R. A. Cross-linked enzyme aggregates: a simple and effective method for the immobilization of penicillin acylase. Org. Lett. 2, 1361–1364 (2000). 10814447 10.1021/ol005593x

[b12] Harmann M. & Kostrov X. Immobilization of enzymes on porous silicas-benefits and challenges. Chem. Soc. Rev, 42, 6277–6289 (2013). 23765193 10.1039/c3cs60021a

[b13] Kumar V. V., Sivanesan S. & Cabana H. Magnetic cross-linked laccase aggregates -bioremediation tool for decolorization of distinct classes of recalcitrant dyes. Sci. Total. Environ. 487, 830–839 (2014). 24785303 10.1016/j.scitotenv.2014.04.009

[b14] Cui J. D. & Jia S. R. Optimization protocols and improved strategies of cross-linked enzyme aggregates technology: current development and future challenges. Crit. Rev. Biotechnol. 35, 15–28 (2015). 23886350 10.3109/07388551.2013.795516

[b15] Wilson L. *et al.* Encapsulation of crosslinked penicillin G acylase aggregates in lentikats: evaluation of a novel biocatalyst in organic media. Biotechnol. Bioeng. 86, 558–562 (2004). 15129439 10.1002/bit.20107

[b16] Kim M. I. L. *et al.* Crosslinked enzyme aggregates in hierarchically-ordered mesoporous silica: a simple and effective method for enzyme stabilization. Biotechnol. Bioeng. 96, 210–218 (2007). 16986168 10.1002/bit.21107

[b17] Talekar S. *et al.* Preparation of stable cross-linked enzyme aggregates (CLEAs) of NADH-dependent nitrate reductase and its use for silver nanoparticle synthesis from silver nitrate. Catal. Commun. 53, 62–66 (2014).

[b18] Kumar V. V. *et al.* Preparation and characterization of porous cross linked laccase aggregates for the decolorization of triphenyl methane and reactive dyes, Bioresour Tech. 119, 28–34 (2012). 10.1016/j.biortech.2012.05.078 22728178

[b19] Talekar S. *et al.* Novel magnetic cross-linked enzyme aggregates (magnetic CLEAs) of alpha amylase. Bioresour. Technol. 123, 542–547 (2012). 22944488 10.1016/j.biortech.2012.07.044

[b20] Luckarift H. R., Spain J. C., Naik R. R. & Stone M. O. Enzyme immobilization in a biomimetic silica suport. Nat. Biotechnol. 22, 211–213 (2004). 14716316 10.1038/nbt931

[b21] Patwardhan S. V. Biomimetic and bioinspired silica: recent developments and applications. Chem. Commun. 47, 7567–7582 (2011). 10.1039/c0cc05648k 21479320

[b22] Luan P. P. *et al.* Chitosan-mediated formation of biomimetic silica nanoparticles: An effective method for manganese peroxidase immobilization and stabilization. J. Biosci. Bioeng. 118, 575–582 (2014). 24913823 10.1016/j.jbiosc.2014.05.003

[b23] Kristensen, J. B. *et al.* Biomimetic silica encapsulation of enzymes for replacement of biocides in antifouling coatings. Green. Chem. 12, 387–394 (2010).

[b24] Kuan, I. C. *et al.* Stabilization of D-amino acid oxidase from *Rhodosporidium toruloides* by encapsulation in polyallylamine-mediated biomimetic silica. Biochem. Eng. J. 49, 408–413 (2010).

[b25] Sun, Q. Y. *et al.* Green and efficient conversion of CO_2_ to methanol by biomimetic co-immobilization of three dehydrogenases in protamine-templated titania. Ind. Eng. Chem. Res. 48, 4210–4215 (2009).

[b26] Forsyth C., Yip T. W. S. & Patwardhan S. V. CO_2_ sequestration by enzyme immobilized onto bioinspired silica. Chem. Commun. 49, 3191–3193 (2013). 10.1039/c2cc38225c 23247081

[b27] Jiang Y. J. *et al.* Preparation of robust biocatalyst based on cross-linked enzyme aggregates entrapped in three-dimensionally ordered macroporous silica. ACS Appl. Mater. Interfaces. 6, 2622–2628 (2014). 24484443 10.1021/am405104b

[b28] Wang, M. F. *et al.* Porous-CLEAs of papain: Application to enzymatic hydrolysis of macromolecules. Bioresour. Tech. 102, 3541–3545 (2011). 10.1016/j.biortech.2010.08.120 20863695

[b29] Zhou Z., Taylor R. N., Kullmann K. S., Bao H. X. & Hartmann M. Mesoporous organosilicas with large cage-like pores for high efficiency immobilization of enzymes. Adv. Mater. 23, 2627–2632 (2011). 21480398 10.1002/adma.201004054

[b30] Abdullah A., Sulaiman N. & Kamaruddin A. Biochemical biocatalytic esterification of citronellol with lauric acid by immobilized lipase on aminopropylgrafted mesoporous SBA-15, Biochem. Eng. J. 44, 263–270. (2009)

[b31] Garcia-Galan C., Berenguer-Murcia A., Fernandez-Lafuente R. & Rodrigues R. C. Potential of different enzyme immobilization strategies to improve enzyme performance. Adv. Syn. Catal. 353, 2885–2904 (2011).

[b32] Gadioua R. *et al.* Temperature-programmed desorption as a tool for quantification of protein adsorption capacity in micro- and nanoporous materials. Colloids Surf, B: Biointerfaces. 73, 168–174 (2009). 19535230 10.1016/j.colsurfb.2009.05.012

[b33] Zhou Zh. & Hartmann M. Progress in enzyme immobilization in ordered mesoporous materials and related applications. Chem. Soc. Rev. 42, 3894–3912 (2013). 23570038 10.1039/c3cs60059a

[b34] Zhang S. & Cui J. D. Enhancement of phenylalanine ammonia lyase production from *Rhodotorula mucilaginosa* by optimization of culture conditions in batch and fed-batch. Biotechnol. Biotec. Eq. 26, 3418–3424 (2012).

[b35] Cui J. D., Zhang S. & Sun L. M. Cross-linked enzyme aggregates of phenylalanine ammonia lyase: novel biocatalysts for synthesis of L-phenylalanine. Appl. Biochem. Biotechol. 167, 835–44 (2012). 10.1007/s12010-012-9738-0 22622644

[b36] Cui J. D., Sun L. M. & Li L. L. A simple technique of preparing stable CLEAs of phenylalanine ammonia lyase using co-aggregation with starch and bovine serum albumin. Appl. Biochem. Biotechol. 170, 1827 (2013). 10.1007/s12010-013-0317-9 23754561

[b37] Li Y. *et al.* Pore size of macroporous polystyrene microspheres affects lipase immobilization. J. Mol. Catal. B-Enzym, 66, 182–189 (2010).

