# Supplementary material for: Mesoporous CLEAs-silica composite microparticles with high activity and enhanced stability
Source: Sci Rep. 2015 Sep 16;5:14203. doi: 10.1038/srep14203 (PMC4570996; doi:10.1038/srep14203)
Supplement: Supplementary Information [file srep14203-s1.pdf]

Supplementary Information:

Mesoporous CLEAs-silica composite microparticles with high activity and enhanced stability

Jiandong Cui[1, 2, 3*] Shiru Jia [3] Longhao Liang [1] Yamin Zhao [1] Yuxiao Feng [1]

[1]Research Center for Fermentation Engineering of Hebei, College of Bioscience and Bioengineering, Hebei University of Science and Technology, 26 Yuxiang Street, Shijiazhang 050018, P R China

[2]Tianjin Key Laboratory of Food-Biotechnology, Tianjin University of Commerce, Beichen district, Tianjin 300134, P R China

[3]Key Laboratory of Industry Microbiology, Ministry of Education, Tianjin University of Science and Technology, 29 Thirteenth Street, Tai Da Development Area, Tianjin 300457, P R China

[*] Corresponding authors:

Jiandong Cui, E-mail: cjd007cn@163.com, Tel: +86-311-81668486

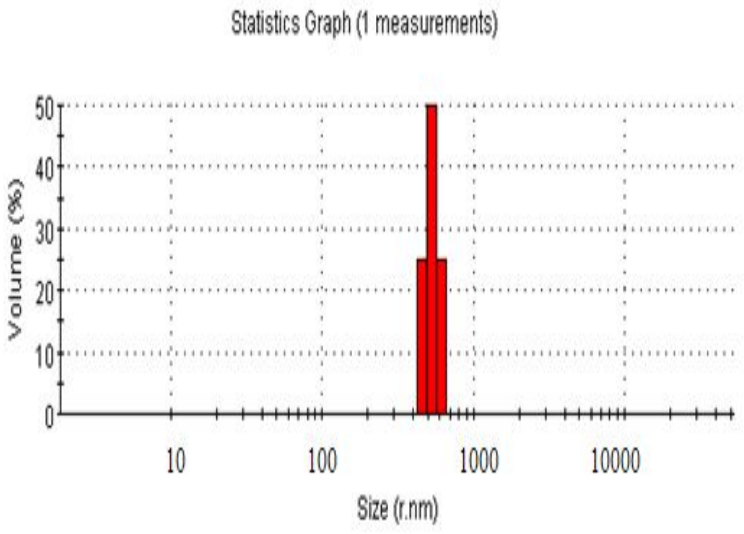

Figure S1. DLS size-distribution diagram of P-CLEAs-Si.

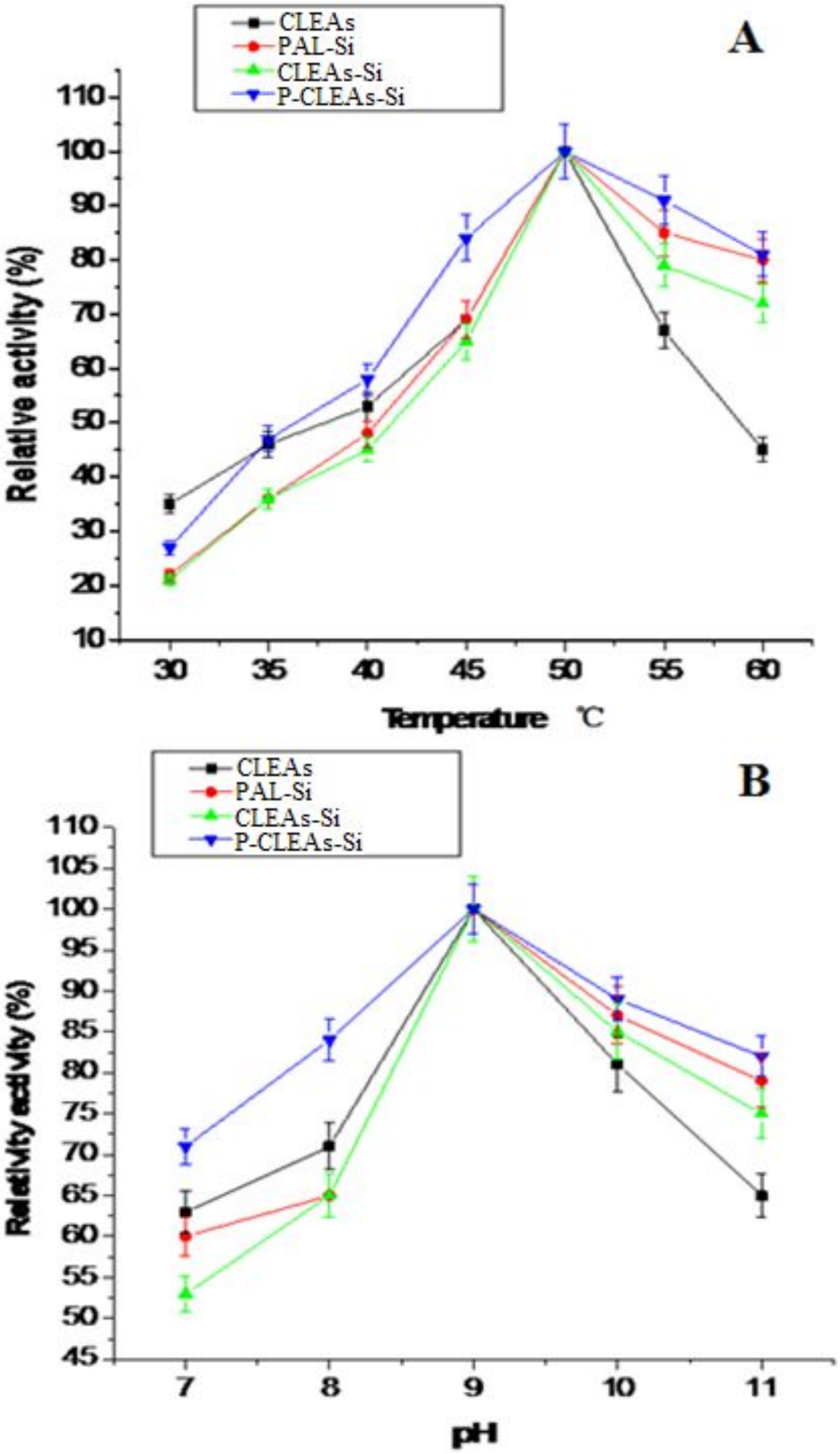

Figure S2. Effect of temperature (A) and pH (B) on the activity of all immobilized PAL; error bars show standard deviations for triplicate measurements.

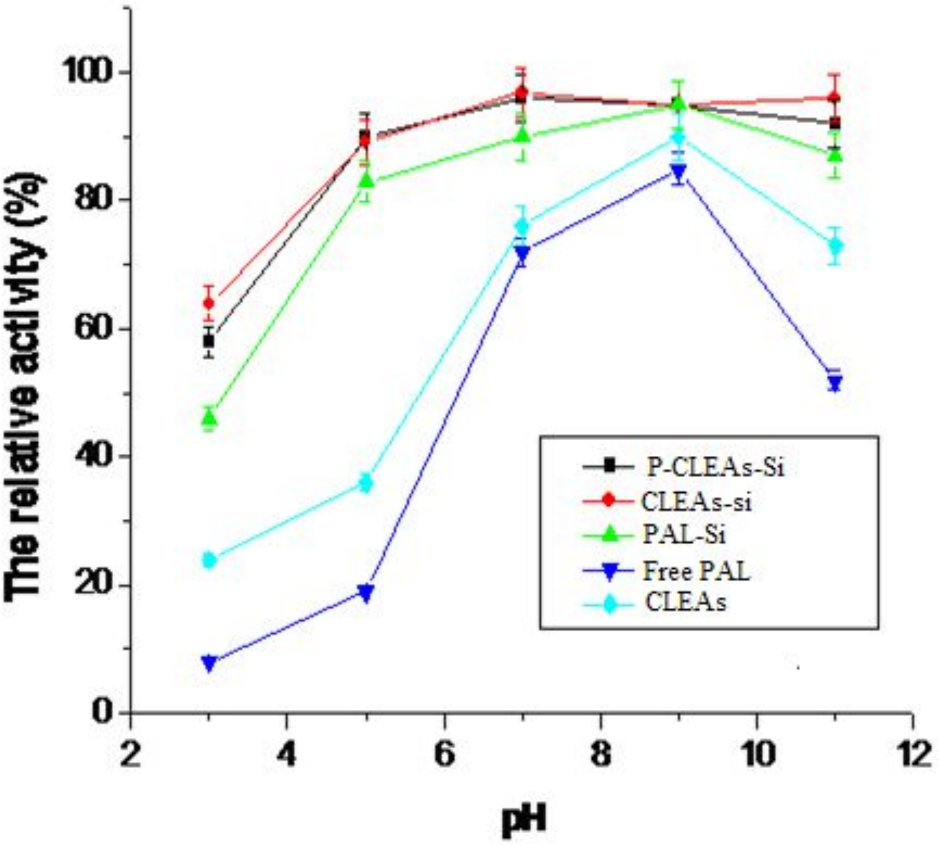

Figure S3. Stability of free PAL, CLEAs, PAL-Si, CLEAs-Si, and P-CLEAs-Si against pH; error bars show standard deviations for triplicate measurements.

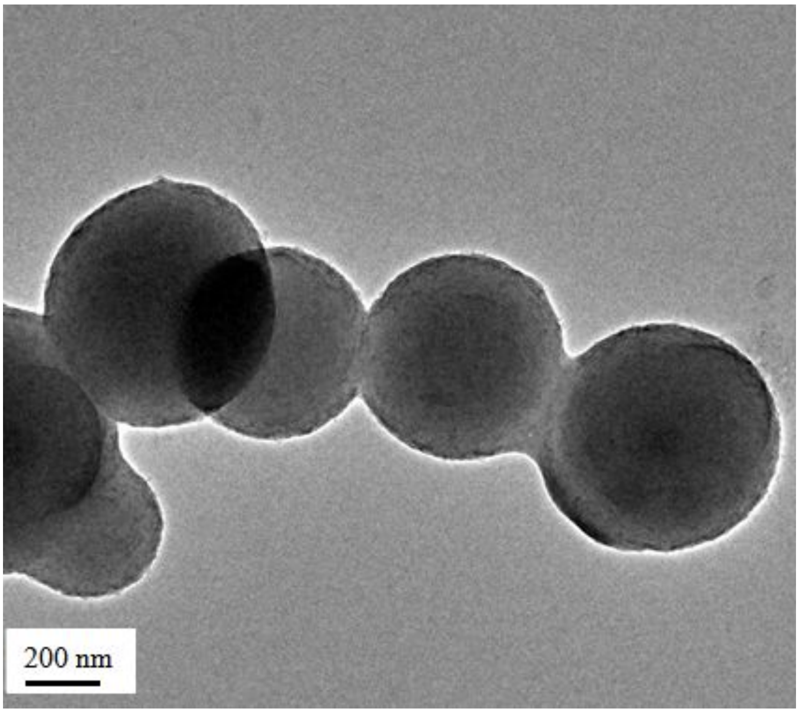

Figure S4. TEM image of P-CLEAs-Si sample after 10 days of shaking.

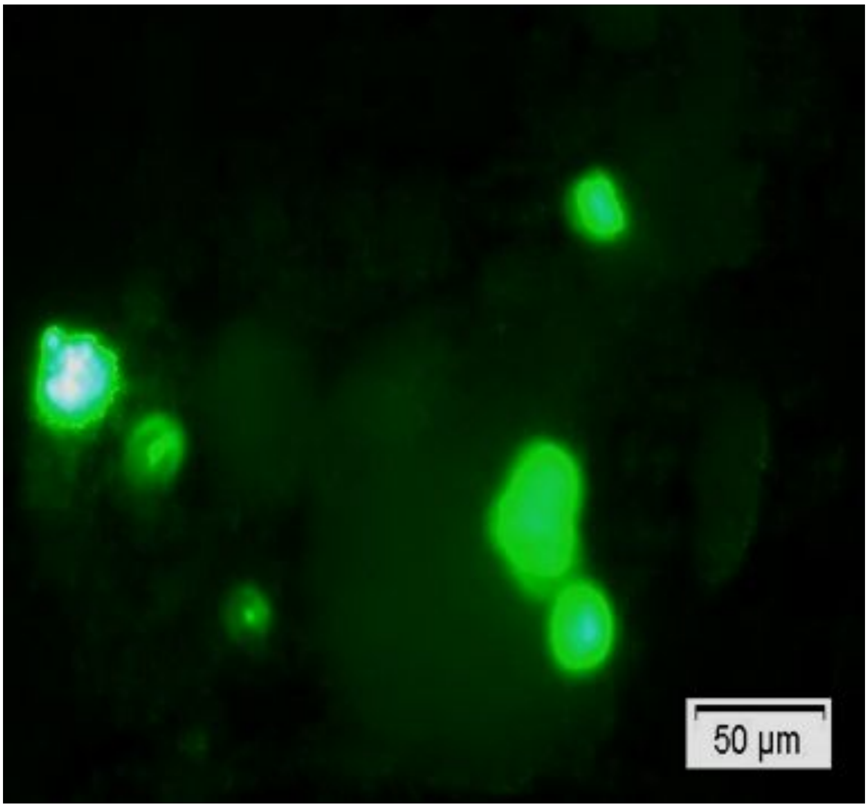

Figure S5. LCSM image of P-CLEAs-Si sample after 10 days of shaking.
